# Additively manufactured micro-lattice dielectrics for multiaxial capacitive sensors

**DOI:** 10.1126/sciadv.adq8866

**Published:** 2024-10-04

**Authors:** Arielle Berman, Kaiwen Hsiao, Samuel E. Root, Hojung Choi, Daniel Ilyn, Chengyi Xu, Emily Stein, Mark Cutkosky, Joseph M. DeSimone, Zhenan Bao

**Affiliations:** ^1^ Department of Mechanical Engineering, Stanford University, Stanford, CA 94305, USA.; ^2^ Department of Materials Science and Engineering, Texas A&M University, College Station, TX 77840, USA.; ^3^ Department of Chemical Engineering, Stanford University, Stanford, CA 94305, USA.; ^4^ Department of Materials Science and Engineering, Stanford University, Stanford, CA 94305, USA.

## Abstract

Soft sensors that can perceive multiaxial forces, such as normal and shear, are of interest for dexterous robotic manipulation and monitoring of human performance. Typical planar fabrication techniques have substantial design constraints that often prohibit the creation of functionally compelling and complex architectures. Moreover, they often require multiple-step operations for production. Here, we use an additive manufacturing process based on continuous liquid interface production to create high-resolution (30-micrometer) three-dimensional elastomeric polyurethane lattices for use as dielectric layers in capacitive sensors. We show that the capacitive responses and sensitivities are highly tunable through designs of lattice type, thickness, and material-void volume percentage. Microcomputed tomography and finite element simulation are used to elucidate the influence of lattice design on the deformation mechanism and concomitant sensing behavior. The advantage of three-dimensional printing is exhibited with examples of fully printed representative athletic equipment with integrated sensors.

## Teaser

3D-printed lattices are used as dielectric layers in multidirectional sensors with applications in wearables and robotics.

## INTRODUCTION

Wearable electronic devices that can sense directional forces are essential for a wide range of fields such as robotic grasp manipulation (*1*–*4*), tracking of human performance (*5*–*8*), and health monitoring (*9*–*11*). Robots, like humans, must be able to differentiate normal pressure, shear force, and torsion to avoid excess gripping forces and prevent dropping objects through sufficient slip feedback (*12*). Similar concepts can be used for the analysis of sport performance and safety through the development of sensorized equipment. In this context, soft devices are required to maximize the conformability to the dynamic, curvilinear surfaces of the body and promote comfort for the user (*12*).

Compliant electronic skins, or eSkins, have been developed to detect multiaxial forces using piezoresistive (*13*–*17*), contact-resistive (*4*, *18*, *19*), triboelectric (*20*), magnetic (*21*), and capacitive (*4*, *14*, *22*, *23*) transduction mechanisms. A capacitive sensing scheme is attractive for its high sensitivity, low hysteresis, fast response, and low power consumption (*24*–*26*). Capacitive pressure sensors usually consist of two planar electrodes separated by an insulating dielectric layer. Our group previously put forth the concept of microstructured dielectric layers, such as micropyramids, lines, and domes, to enhance sensitivity and minimize hysteresis relative to nonstructured elastomer dielectrics (*27*, *28*). Adjustment of sensor behavior has been achieved through the rational microengineering of the dielectric structures (*24*, *28*). Existing force vector sensors have used patterned dielectrics that deform in the vertical and horizontal axes. These dielectrics consist of foams (*2*, *29*–*31*), dome microstructures (*1*), and microfluidic channels (*30*). However, variations between thin-film foam-based capacitive sensors are unavoidable because some pores are comparable in size to film thickness, and it is difficult to control pore size and position during fabrication of the dielectric layer.

Making a device with microstructures or microfluidic channels involves multistep fabrication and assembly processes together with a need for precise alignment during lamination. Photolithography and etching processes, which are typically used to make microstructured silicon molds, are time consuming and have constraints on out-of-plane design possibilities. Furthermore, replicating microstructures from these molds can introduce complications from defects due to delamination. These challenges severely restrict the repeatability across many samples and complicate the fabrication of multiaxial sensors. Moreover, these conventional 2D fabrication processes make it challenging to produce nonplanar structures that are desirable for wearable sports technology.

Additive manufacturing of dielectric layers for capacitive triaxial sensors can address some of the above challenges. This approach potentially allows for the rapid construction of intricate three-dimensional (3D) dielectrics with a single step. Digital design and automation together contribute to the range of structures possible and the reproducibility of samples. Extrusion-based additive manufacturing has been used to create soft shear sensors, but these designs did not have microstructures and instead relied on bulk material deformation to change the device output (*17*, *32*, *33*). 3D-printed lattices, in which struts of various arrangements create controlled porosity, have been reported as dielectric layers recently (*34*, *35*). However, these lattices had large features (~10 mm) and simplistic structures. Multimaterial lattice geometries were additively manufactured by direct light processing but were not used for capacitive sensing (*36*). The properties of additively manufactured gyroids, a specific type of lattice structure, under compression and tension were also investigated; however, that work only evaluated its capacitive sensing capabilities under tension, not compression or shear (*37*). While promising initial demonstrations of 3D printing for capacitive sensing have been reported, to achieve the full potential of this approach for wearable applications, improvements are needed in the design of microstructured dielectrics and development of more sophisticated 3D devices with multiple sensors integrated for discrimination of multiaxial forces such as pressure and shear.

**Fig. 1.**
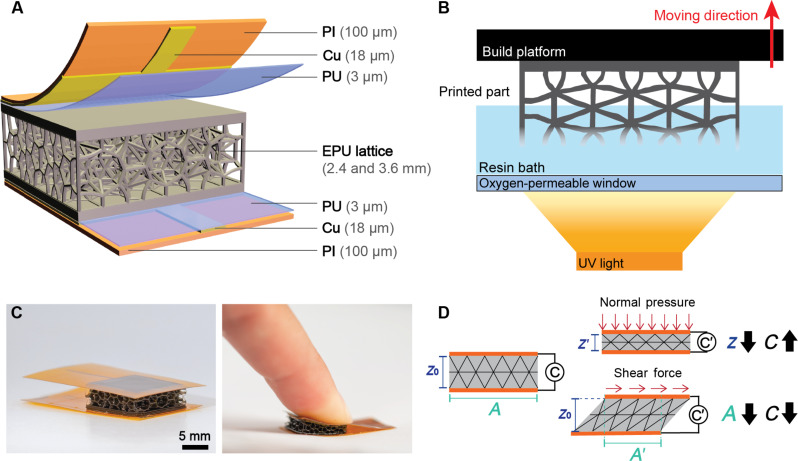
Fabrication and sensing mechanism of single-pixel capacitive sensors based on EPU lattice dielectric layers. (**A**) Schematic showing the copper electrodes on a flexible polyimide (PI) backing that are laminated with a polyurethane thin film to either side of the EPU 40 lattice to form the parallel-plate sensor structure. (**B**) Diagram of CLIP, the additive manufacturing method used to produce the lattice dielectric layer. (**C**) Images of 1 cm by 1 cm flexible capacitive sensor and when pressed by a finger. (**D**) Expected capacitive sensing mechanisms for normal pressure, in which the decrease in vertical separation, *z*, between the two electrodes increases the capacitance and simple shear force, which causes a decrease in overlapping electrode area and a subsequent decrease in capacitance.

Here, we present digitally designed and printed lattice structures for dielectric layers in capacitive pressure and shear sensors (Fig. 1A). An additive manufacturing process that uses a vat of photopolymerizable liquid resin, namely, continuous liquid interface production (CLIP), was leveraged to fabricate samples with readily tunable architecture parameters: (i) lattice type, (ii) thickness, and (iii) volume percentage (Fig. 1B). The effect of these parameters on the sensors’ capacitive response under both normal force and simple shear is reported here. Using the optimized design, we prepared a fully printed device with four capacitive output channels to exhibit the multidimensional force-sensing capability of the lattice. In addition, a sensorized helmet and shoe sole demonstrate the potential for the concurrent printing of lattices and prealigned electrodes with complex geometric designs and multiple capacitive sensing channels toward monitoring external impacts on the head and pressure distribution during walking or running.

## RESULTS

### Design and working mechanism

Our capacitive sensor design uses an elastomeric polyurethane (EPU)–based lattice dielectric layer produced with CLIP. The structure is sandwiched between two flexible electrodes to form a parallel-plate capacitive pressure sensor (Fig. 1C), with a response governed by the equation

$$C=\frac{\varepsilon_{r}\varepsilon_{0}A}{z}$$ (1)

where *C* (farad) is the capacitance, ε*_r_* is the dielectric constant, ε_0_ (farad per meter) is the permittivity of free space, *A* (square meter) is the overlapping area of the electrodes, and *z* (meter) is the distance between the electrodes. If the dielectric constant is unaltered, like in a sensor with a planar dielectric, changes in capacitance are purely geometrically dependent. When normal force is applied to a capacitive sensor, the dielectric compresses, and the distance between the two electrodes decreases, increasing capacitance. When the sensor undergoes simple shear, the *z*-dimension height is constrained to remain constant while the parallel-plate electrodes translate relative to one another. Therefore, the overlapping area between the parallel-plate electrodes, *A*, will decrease leading to a reduction in the capacitance (Fig. 1D). Capacitive pressure sensors are also influenced by microstructuring the dielectric layer, which introduces free air volume to the structure. Because air has a lower dielectric constant than the polymeric component (*38*), the biphasic dielectric layer has a smaller effective ε*_r_* than that of bulk elastomer. Thus, when air is pushed out under compression, the effective dielectric constant—and consequently the capacitance—rises. The micropatterning of the dielectric layer also increases the device sensitivity because the dielectric has improved compressibility because air is more readily displaced at low pressures.

### Fabrication and materials selection

In standard stereolithography-based 3D printing, each subsequent layer of the part becomes adhered to the resin basin and must be mechanically detached before the next layer is exposed. This motion exerts large adhesion forces on the print. CLIP is advantageous in that it has an oxygen-permeable window that inhibits polymerization in the “dead zone” and, thus, does not require mechanical detachment, resulting in 100× increase in fabrication speed and smoothness (*39*, *40*). Hence, CLIP is both significantly faster and able to print more delicate objects. We selected a commercially available EPU resin, EPU 40, for its mechanical properties—great elasticity with a high tear strength—and for its ability to be printed with high resolution. A custom-built CLIP printer with 30-μm print resolution was used to print the 1 cm by 1 cm lattice dielectrics for sensor characterization (*41*, *42*). Each lattice took only about 45 min to print. The EPU 40 lattices required dual curing: (i) ultraviolet (UV) light exposure to form the initial “green” state during printing, and (ii) subsequent baking to induce orthogonal, thermally initiated chemistry to create the material properties. We used a polyurethane interlayer to adhere both electrodes to the dielectric, which formed a single-pixel parallel-plate capacitive sensor (fig. S1). A robust adhesion between the electrodes and the dielectric lattice is important to prevent delamination particularly under high shear forces. A parallel-plate sensor design was chosen for its simple fabrication and straightforward governing equation. In addition, its electric field encompasses the entire lattice compared to the shallow fringe field penetration of an interdigitated capacitor design.

**Fig. 2.**
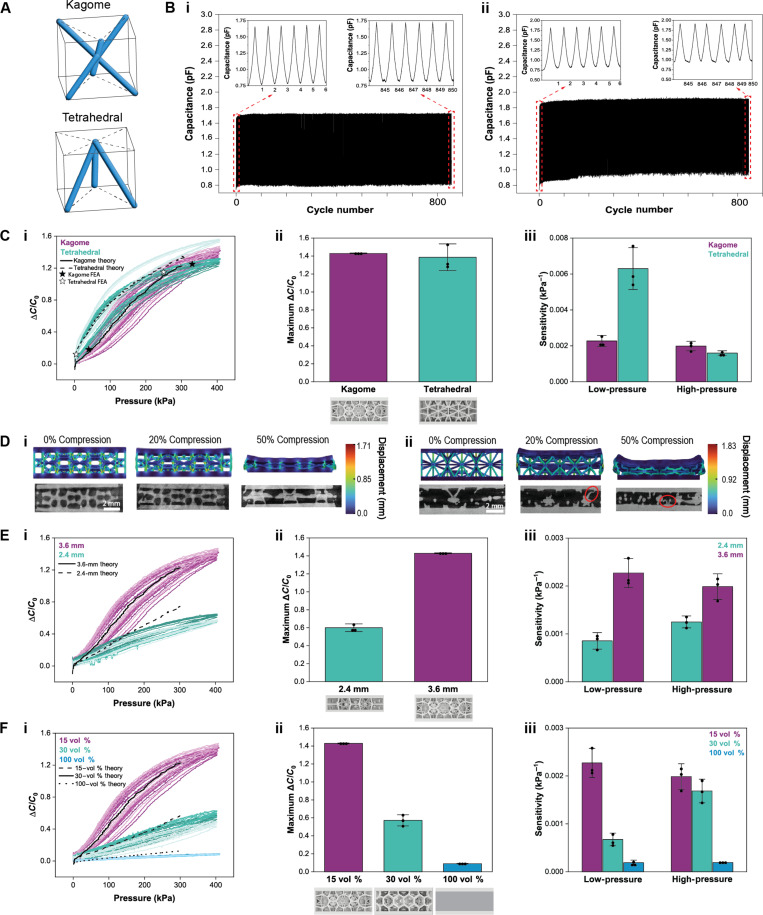
Characterization of lattice dielectric parameters under normal pressure. (**A**) Schematic of single-cell lattice topologies for kagome and tetrahedral. (**B**) Cyclic loading under normal pressure of capacitive sensors fabricated with (i) kagome and (ii) tetrahedral lattice dielectrics. (**C**) Effect of lattice type on sensor output showing (i) experimental Δ*C*/*C*_0_ versus pressure (kilopascal), theoretical output, and FEA results at 20 and 50% strain, (ii) average maximum Δ*C*/*C*_0_ at 400 kPa, and (iii) average low- and high-pressure sensitivity. Error bars are ±SD for *n* = 3. (**D**) Finite element simulation and corresponding representative microCT cross-sectional side view images at 0, 20, and 50% compression for (i) kagome and (ii) tetrahedral lattice metastructures. Buckling of struts in the microCT images can be seen circled in red. The color bar for the finite element model is displacement (millimeter). (**E**) Sensor output due to changing thickness of dielectric layer with (i) experimental Δ*C*/*C*_0_ versus pressure (kilopascal) and theoretical output, (ii) average maximum Δ*C*/*C*_0_ at 400 kPa, and (iii) average low- and high-pressure sensitivity. Error bars are ±SD for *n* = 3. (**F**) Sensing data by changing dielectric volume % between 15, 30, and 100 vol % with (i) experimental Δ*C*/*C*_0_ versus pressure (kilopascal) and theoretical output, (ii) average maximum Δ*C*/*C*_0_ at 400 kPa, and (iii) average low- and high-pressure sensitivity. [B (i)], [E (i)], and [F (i)] show three samples for each parameter set with 3 cycles each. Error bars are ±SD for *n* = 3.

Intentional changes in microstructure were made by varying the lattice’s design (kagome and tetrahedral), vertical thickness (2.4 and 3.6 mm), and volume % (15, 30, and 100 vol %). The kagome and tetrahedral designs were chosen for their distinct single-cell topologies (Fig. 2A) (*43*, *44*). The capacitive sensing response, including (i) initial capacitance (*C*_0_), (ii) maximum relative change in capacitance (Δ*C*/*C*_0_), (iii) low- and high-pressure sensitivity [(Δ*C*/*C*_0_)/Δ*P*] calculated from 0 to 50 and 350 to 400 kPa, respectively, and (iv) normalized hysteresis as a function of the aforementioned lattice parameters are investigated below.

Tight control of metamaterial microstructure (table S1) was needed to achieve reproducibility of capacitive response across devices within the same set. Centrifugal cleaning of the lattices following printing and before the final cure was used to remove the excess resin in the porous lattice structure, which, if cured, would close the voids between the struts. Should this happen, the mechanical properties and resulting capacitive behavior would be greatly affected (fig. S2). Notably, such susceptibility to microstructure established that these lattices are highly tailorable with small changes in lattice formation.

### Characterization of lattice dielectric

#### Normal pressure

The effect of dielectric lattice design, kagome and tetrahedral, on normal pressure was investigated first. The dielectric thickness, volume %, and strut width were printed to be constant at 3.6 mm, 15 vol %, and ~210 μm, respectively, and Δ*C*/*C*_0_ versus pressure (kilopascal) was recorded. When fabricated into single-pixel parallel-plate capacitive sensors as described above, one representative kagome sample showed excellent pressure-sensing stability up to 850 consecutive cycles. When comparing the change in capacitance between the final cycle and that of the initial cycle, there was a 4% change at 400 kPa and <8% change at 0 kPa (Fig. 2Bi). The representative tetrahedral design also displayed prolonged reliability with an ~5% change over 850 cycles at 400 kPa, but there was a 22.6% drift in capacitance at 0 kPa (Fig. 2Bii).

The initial capacitances of three samples each can be seen in fig. S3. When a normal pressure was applied, the discrepancy in percentage change in capacitance between the two designs was not significant (Fig. 2Ci), although tetrahedral dielectrics have a larger SD (Fig. 2Cii). On the loading portion of the curve, the kagome design behaved relatively linearly with no difference in low- and high-pressure sensitivity of ~0.002 kPa^−1^ ± 0.0003 (*n* = 3). However, the tetrahedral design had a greater low-pressure sensitivity of 0.0063 kPa^−1^ ± 0.001 (*n* = 3) than its high-pressure sensitivity of 0.0016 kPa^−1^ ± 0.0001 (*n* = 3) (Fig. 2Ciii). The higher low-pressure sensitivity was likely due to the lower stiffness of the tetrahedral lattice at small displacements, which would allow for more air to be expelled from the dielectric structure, compared to the kagome design, at the same pressure. Microcomputed tomography (microCT) scans were performed under compression to explain the difference in normal compressive behavior between the lattice types. Corresponding finite element analysis (FEA) models with nTopology, which are limited to the linear elastic regime, were produced to qualitatively compare with the microCT results. A quantitative comparison was done between the FEA output and the experimental data at 20 and 50% strain (discussion S1) and is included in Fig. 2Ci. The hexagonal cavity structure formed within the kagome network collapsed under pressure while preserving the integrity of the surrounding struts (Fig. 2Di), making it more difficult to deform. Conversely, the tetrahedral struts began to buckle after only 20% compression, or ~120 kPa of pressure. The tetrahedral lattice’s proclivity for buckling, circled in red in Fig. 2Dii, may be due to its overall longer strut length (*43*), which has an inverse square relation to buckling load according to Euler’s column buckling model (*45*, *46*). The effect of normal pressure strain rates on kagome and tetrahedral lattice–based sensors can be seen in fig. S4A, i and ii, respectively. In both cases, there was minimal rate dependence at low pressures, but the capacitive responses began to diverge around 75 kPa. At loads above this value, strain rate and maximum Δ*C*/*C*_0_ had an inverse relationship. When the force application rate was slower, the viscoelastic polymeric chains making up the lattice likely had a longer time to rearrange, allowing for greater deformation.

Next, we studied 2.4- and 3.6-mm lattice thicknesses (Fig. 2Ei) using a kagome unit structure designed to be 15–vol % EPU. The kagome design was chosen because it deformed less readily under constant mass during fabrication, resulting in better contact, and therefore superior adhesion, to the electrodes. Figure S3B shows that initial capacitance of the lattice dielectric sensors decreased with increasing thickness, which follows the inverse relationship of *C* and *d* in Eq. 1. The higher thicknesses used here compared to thin-film capacitive sensors were constrained to multiples of the lattice unit cell size (1.2 mm) and the need to produce a more 3D structure for reversible compression. In addition, the thicknesses needed to be much greater than the diameter of the lattice struts. It was also evident that a higher lattice thickness had a substantial positive effect on the sensor responsiveness, with Δ*C*/*C*_0_ for the 3.6-mm dielectric more than twofold higher than the 2.4-mm case (Fig. 2Eii). When compared to the apparent constant sensitivity of the 3.6-mm kagome, the 2.4-mm samples had a slight increase in sensitivity at high pressures (Fig. 2Eiii).

Volume percentage was then varied between 15, 30, and 100 vol % while maintaining a kagome lattice design and 3.6-mm-thick dielectric layers (Fig. 2Fi). As expected from eq. S3 in discussion S2, the initial capacitance was the highest for 100–vol %, or solid, dielectric layer (fig. S3C). Lattice volume percentage appeared to have the clearest effect on relative change in capacitance, with a distinct reduction in Δ*C*/*C*_0_ at 400 kPa from 1.43 to 0.57 to 0.09 with an increase in volume % of EPU (Fig. 2Fii). In the low-pressure regime, the sensitivity inversely scaled with volume %. However, under high pressure, the sensitivity plateaued from 15 to 30 vol % but dropped 10-fold for the 100 vol % dielectric (Fig. 2Fiii). The fact that there was no change in sensitivity for lower volume % indicates that the variation of capacitance per unit pressure, as the biphasic medium is compressed at high forces, had reached a steady state despite 15 vol % having had more air overall.

Force versus deformation data was also obtained for each lattice type (fig. S5). The lattice design had relatively minimal effect on normal compressive mechanical properties at large displacements, but decreasing lattice height and increasing lattice volume % both resulted in stiffer structures that more readily opposed applied external pressure. The stiffness of the lattice directly affects the degree to which air is pushed out of the porous medium for a given pressure and therefore determines the capacitive behavior of the sensor. The experimental displacement and force data were then used to calculate the expected capacitive response based on a theoretical model (discussion S2) and is plotted in Fig. 2 (Ci, Ei, and Fi).

The normalized hysteresis of the single-pixel sensors was quantified as the difference between the loading and unloading curve of Δ*C*/*C*_0_ at half of the maximum pressure, in this case 200 kPa, divided by the total Δ*C*/*C*_0_ at maximum pressure (fig. S6). Unexpectedly, the average normalized hysteresis of kagome was found to be 28% greater than that of tetrahedral despite the latter’s lower effective mechanical stiffness and buckling, potentially because of more contacts between adjacent struts during compression (fig. S6A). To ascertain the degree of adhesion of EPU 40 to itself, such as what would occur when neighboring lattice struts come into contact, we performed a 180^o^ peel test (fig. S7A). The resulting calculated interfacial toughness of the self-adhesion of EPU 40 was non-negligible, as it was on the same order of magnitude as that of the adhesion of scotch tape to EPU 40 (fig. S7B). Thus, the persistence of strut sticking as pressure was removed may have prevented the capacitance from returning on its initial loading pathway. The response times to loading and unloading of a 200-mg weight (2 kPa) for kagome and tetrahedral lattices are shown in fig. S8. Both lattices reached their maximum percent capacitance change in ~1.1 s but took 2.58 s to return to the original position after removal of the mass. The delay in unloading supports the hypothesis of strut adhesion.

The inherent viscoelasticity of bulk EPU 40 was studied through the compression of a solid, printed puck on the Instron, which showed non-negligible hysteresis (fig. S9). It is hypothesized that strut adhesion has a larger influence on capacitive hysteresis than bulk viscoelasticity due to the fact that the solid 100–vol % dielectric has a significantly lower hysteresis than 15 and 30 vol %. It can then be concluded that the cause of hysteresis in lattices differs from existing state of the art in which microstructuring dielectrics were shown to decrease hysteresis. Hysteresis may potentially be reduced by using a more elastic polymer for the dielectric layer. Structural engineering could also be leveraged, such as by designing a lattice with a lower elastomeric volume % to reduce contact between adjacent struts or by designing rigid pillars to be printed at the corners of the lattice to assist in the return of the dielectric to its original shape.

#### Shear force

**Fig. 3.**
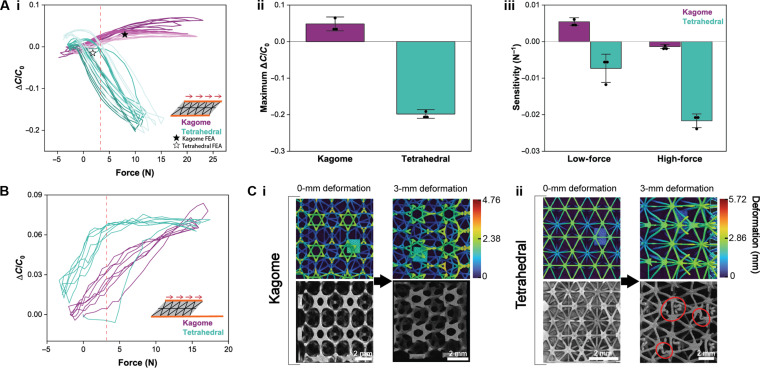
Results of single-pixel lattice dielectric capacitive sensor subjected to simple shear. (**A**) Comparison of lattice type dielectrics. (i) Relative change of capacitance versus shear force (newton) for kagome and tetrahedral with three samples of 3 cycles each and FEA results simulated at 30% strain. (ii) Kagome resulted in an unexpected, positive Δ*C*/*C*_0_ while tetrahedral had a negative response. (iii) Low- and high-force sensitivities of kagome and tetrahedral showed the superior sensing capability of the latter lattice design. Error bars are ±SD for *n* = 3. (**B**) Isolation of the influence of changing dielectric constant by holding electrode overlapping area constant. (**C**) Finite element simulation and the corresponding representative microCT images of (i) kagome and (ii) tetrahedral lattices when subjected to 0- and 3-mm simple shear deformation. Buckling of struts can be seen in tetrahedral within the red circles. The color bar for the finite element model is deformation (millimeter).

The lattice-based sensors were next subjected to shear. With lattice dielectric layers, the relative change in capacitance is a function of two simultaneously changing quantities—the change in overlapping area, *A*, and the effective dielectric constant, ε*_r_*, which is dependent on the structure and biphasic composition of the lattice under deformation. With lattice thickness and volume % held constant, the effect of lattice design was studied again. Notably, kagome and tetrahedral were found to have opposite responses: Δ*C*/*C*_0_ decreased for tetrahedral as anticipated but increased for kagome (Fig. 3Ai). For a mechanistic understanding of this phenomenon, we isolated the competing effects of geometry versus effective dielectric constant by lengthening the bottom electrode such that, upon shearing, *A* will not change. Figure 3B shows that at forces of less than 3 N, the effective dielectric constants of tetrahedral and kagome both increased. Yet, as the force increased, the contribution of ε*_r_* toward Δ*C*/*C*_0_ for tetrahedral plateaued, whereas it continued to increase linearly for kagome. It can thus be concluded that, for forces higher than 3 N on tetrahedral-based sensors, the reduction in *A* dominated, and Δ*C*/*C*_0_ subsequently decreased. Conversely, the continuous increase in ε*_r_* of kagome offset the change in area, resulting in a near-zero capacitive sensitivity at high forces.

Furthermore, analysis of cross-sectional microCT images was used to qualitatively understand the behavior of the two topologies under shear deformation. While the hexagonal structures of kagome underwent a minimal change in form and all struts remained intact (Fig. 3Ci), there was clear evidence of buckling in tetrahedral, as seen in the red circles in (Fig. 3Cii). Under shear, the tetrahedral struts seem to have collapsed onto themselves. This microscale buckling may help explain why the dielectric constant of tetrahedral reached a plateau, as the lattice struts may have simply failed rather than pushing air from the biphasic dielectric. In addition, the weaker structure’s collapse likely contributed to tetrahedral’s lower shear modulus (fig. S10) and, consequently, its larger sensor responsiveness and sensitivity (Fig. 3A, i and ii) when compared to the more robust kagome structure. However, this micromechanical phenomena could not be captured on the corresponding FEA simulations as the program is limited to linear elastic deformation, which does not fully represent the nonlinear mechanical response beyond the elastic limit of the EPU. However, discussion S1 presents a quantitative comparison between the Δ*C*/*C*_0_ values expected on the basis of FEA simulations and those found in experimental studies. This data, simulated at 30% strain, is also denoted in Fig. 3Ai.

When the dielectric thickness was changed, we observed that a thicker lattice resulted in 4.5-fold larger maximum Δ*C*/*C*_0_ (fig. S11A). By comparing different lattice volume %, we saw that 15 vol % had a substantially higher responsiveness than 30 vol % (fig. S11B). It was evident from the sensitivity data that neither 2.4-mm nor 30–vol % kagome dielectric layers had appreciable sensing capabilities. Therefore, using tetrahedral lattice topology in dielectrics was the most effective method for achieving high-quality signal output, and the negative Δ*C*/*C*_0_ behavior may allow for more effective deconvolution via machine learning when multiaxial forces are combined in a single external stimulus (*47*, *48*). These sensitivity results and those due to normal pressure can be compared to existing multiaxial capacitive sensors in table S2.

We then compared the normalized hysteresis of lattices under shear. Between kagome and tetrahedral, the latter was found to have a significantly larger hysteresis (fig. S6B). This result is attributed to the inherent viscoelasticity of the EPU. In this case, we also noticed that buckled struts came into contact with themselves when under shear, as seen in the red circles on the microCT image in Fig. 3Cii, which may have contributed to self-adhesion and caused hysteresis. This is a different phenomenon than the contact of adjacent struts when the kagome lattice was compressed under normal pressure, discussed previously. In addition, the greater hysteresis may also be a result of the slightly higher shearing rate to which tetrahedral was subjected. The influence of different shear rates on the capacitive response of a tetrahedral lattice sensor, which was superior for shear sensing because of its greater change in capacitance and relative linearity at high pressures, can be found in fig. S4B. Minimizing the rate dependence of polymeric sensors is a grand challenge for the field of soft electronics. Changing lattice thickness had no effect on hysteresis. However, unlike the behavior under normal pressure, increasing volume % of the lattice increased normalized hysteresis during shearing. With a higher density of struts, it would be expected that there would be more buckling-induced contacts and, thus, a greater degree of EPU self-adhesion.

### Fully printed multidirectional sensor

**Fig. 4.**
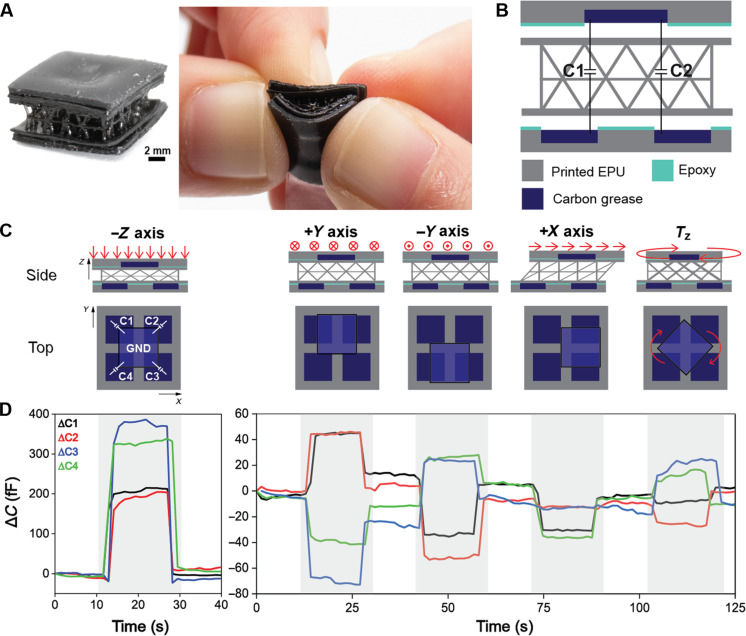
A fully printed, flexible lattice-based multiaxial capacitive sensor. (**A**) Images showing components and completely fabricated sensor. (**B**) Schematic of sensor body, including two covers and the lattice dielectric, carbon grease electrodes, and epoxy adhesive. (**C**) Representation of applied loading in four directions and the resulting movement of the ground electrode from a side and top view. (**D**) Experimental data corresponding to the forces in (C).

The practicality of additively manufacturing capacitive pressure sensors was demonstrated by printing a high-resolution, flexible multiaxial sensor. We printed a three-component design (Fig. 4A) including an EPU tetrahedral lattice, a bottom lid with four cavities for the sensing electrodes, and a top lid with one cavity as the common ground. These cavities were then filled with carbon grease and adhered together with a thin layer of epoxy (Fig. 4B). Carbon grease was chosen as the electrode material for its conductivity and high viscosity, such that it would not seep when the sensor was subjected to high normal pressures. This multiaxial device design is advantageous over state of the art in its considerable minimization of fabrication steps and alignment variation during lamination (*2*, *30*). Figure 4C shows the electrode layout design, and the shifts in the four capacitive channels can then be compared to distinguish between normal pressure, shear force, and torsion. The experimental change in capacitance of the device under various deformation types can be seen in Fig. 4D. The directionality of applied forces can be ascertained using the capacitive governing relationship in Eq. 1. Under normal pressure, the overlapping area, *A*, between the ground and sensing electrodes did not change. However, the distance, *d*, between them decreased, which led to an increase in capacitance of C1, C2, C3, and C4. When the fully printed sensor was subjected to shear along the positive *y* axis, the ground electrode moved over sensing electrodes 1 and 2. This change in overlapping area resulted in an increase in capacitances C1 and C2 and a simultaneous decrease in C3 and C4. Conversely, when the ground electrode was sheared in the negative *y* axis, the overlapping area between the ground electrode and electrodes 3 and 4 increased, while overlapping area with 1 and 2 decreased. As a result, C3 and C4 had a positive change in capacitance while C1 and C2 had a negative change in capacitance. When simple shear in the positive *x*-direction was applied, C1 and C4 decreased as expected because of a smaller overlapping area. It is noteworthy, though, that C2 and C3 had no change. This unexpected result could be due to anisotropy in the lattice dielectric, leading to changes in effective ε*_r_* that counters the influence of the change in area. Last, clockwise torsion could be detected through the increase of capacitance of channels 3 and 4 and a decrease in channels 1 and 2, but to a lesser extent than that of the negative *y*-direction. The capacitive changes under *T*_z_ can be explained by changes in orientation of the ground electrode with respect to the four sensing electrodes. Differentiation between various external forces can be qualitatively achieved by comparing the capacitive outputs of the four channels as the ground electrode moves tridirectionally. Machine learning would be required to discriminate more complex actions, such as combined loadings (*47*, *48*).

### Sensorized sports apparatuses toward human performance tracking

**Fig. 5.**
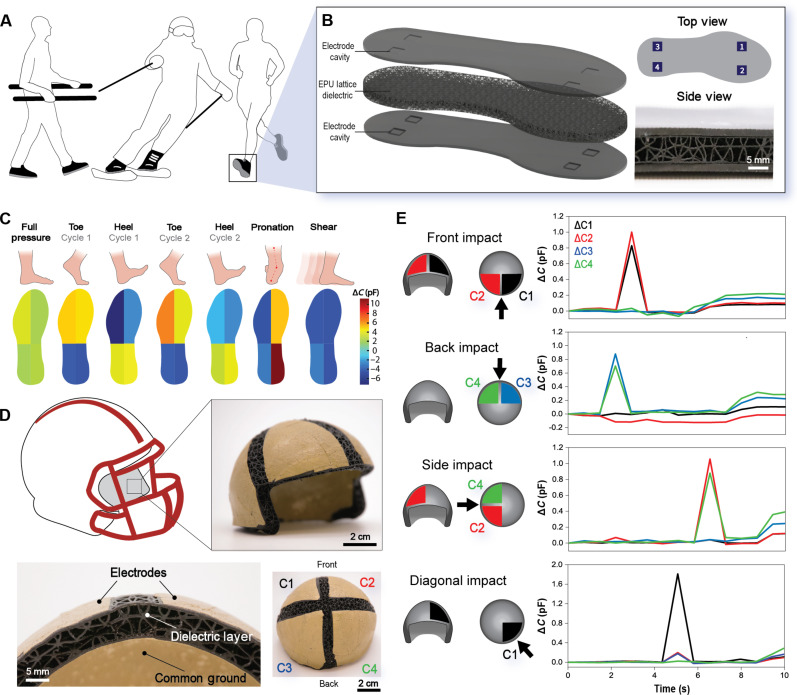
Demonstrations of applications for lattice-based sensing capabilities. (**A**) Potential use cases for a sensorized in-sole include gait tracking during rehabilitation and athletics. (**B**) Computer-assisted design rendering of the shoe sole layers. The top view indicates the four capacitive outputs. The side view is an image of the final device. (**C**) Experimental measured change in capacitance (Δ*C*) results of the four quadrants to different pressure applications. Color bar is in picofarads. (**D**) Miniature helmet lining representing the cushioning inside a football helmet with four capacitive sensing electrodes (C1 to C4) and a common ground made from thin composite films of PPG-HB and silver flakes. (**E**) Experimental measured change in capacitance when the model helmet lining is struck from different directions with a solid, nonconductive object.

We further demonstrated the utility of CLIP for advancing toward more complex sensor systems by fabricating representative sensorized athletic equipment. Soft, elastomer-based wearables are desirable for their amenability to large physical movements, comfort for the user, and impact absorption capability. First, a three-component latticed insole was printed with two parallel-plate sensors under the ball of the foot and two under the heel. Such a concept would serve as a method for in situ human gait tracking in orthotics, rehabilitation, or athletic contexts, as well as characterizing stance or weight distribution in sports like skiing (Fig. 5A). As above, the electrode cavities were built into the cover design and filled with carbon grease (Fig. 5B). We showed that when a person stood on the intrinsically sensorized shoe sole and shifted their weight, a change in capacitance indicated the applied pressure and corresponding quadrant of the foot. Full pressure, 2 cycles of toe and heel pressure, pronation, and shear slip were studied. Activation of a region of the foot was seen when the capacitance increased because of the compression of the lattice dielectric and resulting approach of the two electrodes. Conversely, the inactive regions experienced negative capacitance changes, likely because of the expansion of the metamaterial separating the electrodes. In the case of shear slip of the foot, all quadrants showed negative output due to the reduced overlapping area of the electrodes as the top of the insole slid backwards relative to the bottom (Fig. 5C).

Building upon this concept, we produced a small-scale tetrahedral latticed helmet lining with a diameter of ~7 cm. A conductive composite of a self-healing polyurea based on a poly(propylene glycol) backbone linked by mixed strength hydrogen bonding units (PPG-HB) with a 1:1 loading by mass of silver flakes (1 to 10 μm) was hot pressed into thin films for the electrodes (*49*) (Fig. 5D). This composite electrode material was chosen for its self-healing ability that allowed multiple pieces of film to bond together into an electrode covering a larger area. Furthermore, the composite’s heat processability eliminated the need for volatile solvents, and it had a robust bond to EPU following annealing. By using four sensing electrodes and a common ground, we were able to discern the directionality of an impact with a solid, nonconductive material by measuring which channels were activated, and the scale of the impact is represented by the degree to which the capacitance changes (Fig. 5E). Once this apparatus can be scaled up, it may have potential use in full-scale helmets as a continuous cranial collision monitoring approach for concussion prevention (*5*).

## DISCUSSION

We described the use of an additive manufacturing CLIP process to print high-resolution digitally designed micro-lattice dielectric layers for normal and shear capacitive sensing. This facile fabrication approach was leveraged to produce versatile metastructures with distinct capacitive responses, which would be too delicate to manufacture on common 3D-printing platforms. The salient advantages of CLIP, compared to conventional planar fabrication strategies, is its ability to produce volumetric flexible structures and easily modifiable, complex microarchitectures. In our characterization experiments and demonstrations, we used three electrode categories—thin metal on a flexible substrate, fluidic carbon grease, and a thermoplastic elastomeric composite. Their utility spans the continuum from parallel-plate sensors with simple multiaxial deformations to protuberant surfaces and complex geometries for which the material needed to be easy to cut to the desired shape and highly compliant. To improve the pliability of the entire device, electrode material selection can be expanded to include conductive polymers or spray-coated high–aspect ratio nanomaterials, and the dielectric lattice may be printed from a lower-modulus elastomer. We envision a future in which 3D body-scanning technology can be combined with digital design and additive manufacturing to create personalized health monitoring wearables, such as for concussion tracking or exoskeletons, which can be specifically designed to match the curved surfaces of the body and fabricated in a single step. Expected challenges to achieve this goal may include multimaterial printing, scaling up lattice area without compromising resolution, and decreasing device thickness while maintaining shear sensitivity. Soft, 3D-printed lattices dielectric layers have the potential to meet the multipurpose needs of capacitive multiaxial sensing systems for wearable devices.

## MATERIALS AND METHODS

### Single-pixel lattice printing and cleaning

EPU (EPU 40), acquired from Carbon Inc. was used for printing single-pixel lattices. The design parameters of the lattices can be found in table S1. The single-pixel lattice was designed in the Carbon Engine Design Software. Before use, EPU 40 was pretreated upon receipt by heating to 40°C for 3 hours and then cooled to room temperature. The EPU 40 was then dispensed through a carbon mixing dispenser gun directly into the home-built 3D printing vat. All single-pixel lattice dielectric layers were printed at 120-ms initial exposure time under a UV intensity of 18.82 mW/cm^2^ and 80-ms exposure time per layer under UV intensity of 12.55 mW/cm^2^. Upon completion of printing, the single-pixel lattices underwent an optimized cleaning process. First, they were rinsed with isopropyl alcohol (IPA; Thermo Fisher Scientific) for 30 s and subjected to centrifugation for 4 min at 400 rpm. They were then rinsed in IPA for another 30 s and centrifuged for 2 min at 600 rpm. Last, the lattices were baked at 120°C for 8 hours and cooled to room temperature before proceeding with sensor fabrication.

### Single-pixel sensor fabrication

The flexible electrodes were ordered from PCBWay. The polyimide substrate was 100 μm thick with 18-μm copper electrodes patterned on top. 3M copper tape was used as the connectors and affixed to the copper electrode using MG Chemicals 8331D Silver Conductive Epoxy. The interconnect was then sealed with Loctite EA 1C epoxy. A solution (100 mg/ml) of Channel Prime Alliance Elastollan 1185A10V thermoplastic polyurethane elastomer in cyclohexanone was then used for the adhesion layer using 0.5 ml spin coated at 1500 rpm and an acceleration of 1500 rpm/s for 30 s. The photocured lattice was then placed onto the electrode and cocured at 70°C for 1 hour. The adhesion layer was then applied to the second electrode, and the curing process was repeated to create a parallel-plate structure.

### Normal pressure testing

A rigid applicator was placed on top of the device for even distribution of force. A Mark-10 Series 5, Force Gauge Model M5-10 was used to measure the exerted loads as an automated vertical stage moved upward. Capacitance values were measured with an Agilent E4980A Precision LCR Meter at 10 kHz and 5 V. All sensors were tested with 5 cycles of 40 N with a 5-μm step size at a 0.06 mm/s strain rate. The normal pressure testing apparatus is shown in fig. S12A.

### Shear force testing

The top of each sensor was affixed to a motorized horizontal linear stage to apply shear. The bottom was affixed to an ATI Gamma 6-Axis Force and Torque load cell, which was used to measure the applied shear force. Each device was displacement controlled to reach an approximate shear force of 20 N. Kagome was laterally deformed 4 mm, and tetrahedral was deformed 5 mm with a step size of 5% the total displacement, resulting in shear rates of 0.15 and 0.19 mm/s, respectively. The Agilent E4980A Precision LCR Meter at 10 kHz and 5 V was used to measure capacitance. A schematic can be found in fig. S12B.

### FEA simulation

The FEA was conducted using nTopology software. First, the lattice design STL files were imported as a mesh, converted into a voxel grid (voxel size, 0.2), underwent a surface remesh (edge length, 1; span angle, 30; growth rate, 2; and feature angle, 45), and then a volume remesh (edge length, 2 and regularity factor 10). The mesh was then converted to implicit body and transformed into a CAD body. To set up the mesh for FEA, the tetrahedral mesh was converted into finite element volume mesh, and the FEA parameters were input as follows for simulations: kagome (compression: Young’s modulus of 40 kPa, maximum applied force 40 N; shear: maximum applied force of 20 N) and tetrahedral (compression: Young’s modulus of 40 kPa, maximum applied force of 40 N; shear: maximum applied force of 15 N). These applied forces were simulated as distributed across the entire 1-cm-wide top surface of the model. Detailed simulation parameters can be found in discussion S1.

### microCT characterization

The microCT characterization was conducted using SkyScan microCT, Bruker, MA with a rotation step of 0.2°, 360° of scanning, and a minimum scan resolution of 3.6 μm. A compression apparatus was designed with top and bottom acrylic plates (4.5 mm by 4.5 mm) held together with adjustable screws to control the compression height on the single-pixel lattice. The single lattices were first placed inside the apparatus, and the compression distances were adjusted through tightening the screws to reach the desired displacement. Once the height was verified with a caliper, the screws were fixed in place with nuts. The entire compression apparatus containing the lattice was then subjected to microCT scans to obtain direct observation of lattice cross-sectional deformation under normal compression.

The shear apparatus was designed similarly with top and bottom acrylic plates (6 mm by 6 mm) held together with adjustable screws. The top and bottom of the lattice dielectric were then epoxied to each plate. The acrylic top plate had screw holes with known intermediate distances to allow for adjustment to the desired shear displacement. Last, the shear apparatus containing the lattice was subjected to microCT scans to obtain direct observation of lattice cross-sectional deformation when subjected to simple shear.

### Adhesion testing

Interfacial toughness was measured using the ASTM D903 180^o^ peel test. Strips of EPU 40 were printed to be 155 mm by 17.81 mm by 2 mm. All tests were conducted with a strain rate of 152.4 mm/min. Interfacial toughness was calculated by doubling the average force plateau and dividing by the sample width (*50*).

### Compression testing

Compression testing of a bulk printed EPU 40 was performed following ASTM D575-91. A puck of diameter 28.6 mm and a height of 12.5 mm was tested on an Instron 5565. Three samples were tested for 5 cycles at a controlled total displacement of 2 mm with a compression rate of 12 mm/min.

### Fabrication of multidirectional sensor

The three components (top lid with ground electrode cavity, bottom lid with four sensing electrode cavities, and the lattice dielectric) were manufactured with EPU 40 resin on a custom-built CLIP printer. The lattice was printed at layer height of 10 μm and a UV intensity of 56.15 mW/cm^2^ per layer, with an exposure time of 165 ms and a dark time of 2 s. The printed lattices were cleaned by rinsing with IPA for 30 s, centrifugation for 4 min at 400 rpm, a second rinsing with IPA for 30 s, and centrifugation for 2 min at 600 rpm.

The five cavities of the fully printed multidirectional sensor were filled with MG Carbon Conductive Grease and connected to 3M copper tape leads. The sensor was compressed to its maximum with a nonconductive object while measuring the individual capacitance values of each channel successively with an Agilent E4980A Precision LCR Meter at 10 kHz and 5 V. The sensor was then adhered to the base of a square jig created on a Formlabs 2 3D printer with Clear Resin V4 to set a constant sensor height. An acrylic rectangle was laser cut to size and mounted to the top of the sensor but was otherwise free floating. An identical sequence of directional translations was made four times while measuring in the same manner as described above.

### Printing of sensorized sports apparatuses

The sensorized insole and helmets were fabricated as follows. For both designs, the solid body was created in Solidworks and latticed as a tetrahedral unit cell with 5.5-mm cell size and 0.66-mm strut diameter. A 0.5-mm-thick skin was added to the lattice in Fusion 360 to help with electrode adhesion. The parts were then printed in EPU 40 on the commercially available Carbon M2 printer using automated printing parameters and 50-μm layer height. The parts were then postprocessed by first spraying in dipropylene glycol methyl ether for 7 min, followed by resting the part on its side for 5 min to allow excess uncured resin to drain. This was followed by rinsing in IPA for 30 s and gentle air blasting for another 30 s, repeated as necessary. The parts were then thermally cured at 120°C for 8 hours.

### Sensorized sports apparatus fabrication and testing

For the shoe sole, the cavities in the top and bottom covers were filled with MG Carbon Conductive Grease, and 3M copper tape was used as leads. The covers were then adhered to either side of the lattice dielectric layer.

For the helmet lining, a self-healing polyurea based on a PPG-HB with a 1:1 loading by mass of silver flakes (1 to 10 μm) was processed for use as an electrode (*49*). It was hot pressed at 130°C to a film ~70 μm thick (Fig. 5D). The electrodes were cut to shape and pressed onto the EPU 40 helmet lining, A large single electrode was placed onto the interior surface to serve as a common ground, and a single electrode was positioned on each quadrant of the exterior surface to create four capacitive sensing pixels. The helmet lining and electrodes were then annealed overnight at 70°C to allow the dynamic polymer to conform to the shape of the helmet and make a robust interface between the two polymers. Copper tape (3M) leads were then adhered to the five electrodes using MG Chemicals 8331D Silver Conductive Epoxy. A spherical mount was printed on a FormLabs2 with Clear V4 resin. The helmet lining was placed onto the mount and affected with a nonconductive object.

In both demonstrations, a Texas Instruments FDC1004 four-channel capacitance-to-digital converter was used using a common-ground configuration. The digital signal was logged using an Arduino Uno microcontroller. The sampling frequency to collect all four capacitances was 1.4 Hz.
