# Supplementary material for: Additively manufactured micro-lattice dielectrics for multiaxial capacitive sensors
Source: Sci Adv. 2024 Oct 4;10(40):eadq8866. doi: 10.1126/sciadv.adq8866 (PMC11451511; doi:10.1126/sciadv.adq8866)
Supplement: Supplementary file 1 — Supplementary Text Figs. S1 to S12 Tables S1 and S2 [file sciadv.adq8866_sm.pdf]

Science Advances
AAAS

Supplementary Materials for

Additively manufactured micro-lattice dielectrics for multiaxial capacitive sensors

Arielle Berman *et al.*

Corresponding author: Joseph M. DeSimone, jmdesimone@stanford.edu; Zhenan Bao, zbao@stanford.edu

Sci. Adv. **10**, eadq8866 (2024)
DOI: 10.1126/sciadv.adq8866

This PDF file includes:

Supplementary Text
Figs. S1 to S12
Tables S1 and S2

Supplementary Text

Discussion S1

Our FEA model based in nTopology cannot simulate the nonlinear stress-strain response of our material. However, by obtaining the gradient of the stress (σ) and strain (ε) at the given strain (ε), we can use our experimental data to estimate the Young's modulus ($E = \frac{\sigma}{\varepsilon}$) at the given strain ($\varepsilon$) using a +/- 0.03% (strain) difference window. Due to the nonlinear stress-strain response of our material, the experimentally extracted Young's modulus (E) at low strain (20%) and high strain (50%) used for approximating our material's response are therefore also different. The extracted Young's modulus were used to run the FEA simulation to obtain the corresponding displacement, stress, and capacitance responses for both shear and compression deformation. Our approximation showed that the predicted capacitance responses are close to our experimental results. The results are summarized in the following tables:

Compression: comparison between FEA and Experiment capacitance response

**Design**	**Strain**	**Young's modulus (MPa)**	**Displacement (mm)**	**Stress (kPa)**	$\Delta C/C_0$ **(FEA)**	$\Delta C/C_0$ **(Expt)**
Kagome 15% 3.6mm	20%	0.54	0.94 mm	40	0.18	0.27
Kagome 15% 3.6mm	50%	2.78	1.54 mm	330	1.25	1.25
Tetrahedral 15% 3.6mm	20%	0.14	0.66 mm	5	0.12	0.14
Tetrahedral 15% 3.6mm	50%	2.54	1.83 mm	250	1.15	1.17

Shear: comparison between FEA and Experiment capacitance response

**Design**	**Strain**	**Young's modulus (MPa)**	**Displacement (mm)**	**Stress (kPa)**	$\Delta C/C_0$ **(FEA)**	$\Delta C/C_0$ **(Expt)**
Kagome 15% 3.6mm	30%	0.70	2.9 mm	80	0.029	0.031
Tetrahedral 15% 3.6mm	30%	0.38	3.2 mm	18	-0.015	-0.056

We found that the differences between FEA simulated capacitance response and experimentally obtained capacitance response are greater under small strain. In addition, the shear capacitance response showed greater differences in comparison to compression capacitance response. It is worth mentioning that our current comparison is only an approximation of a non-linear stress-strain response of our viscoelastic latticed material.

Discussion S2

The dielectric constant, ε_r, of the lattice structure in Eq. (1) can be represented as a function of the initial lattice height, z_0, current lattice height under compression, z, the initial volume percent of the material, φ_0, and the dielectric constants of EPU and air, $\varepsilon_{EPU} = 4$ and $\varepsilon_{air} = 1$, respectively:

$$\varepsilon_r = \left(1 - \varphi_0\left({}^{z_0}/_{z}\right)\right)\varepsilon_{air} + \varphi_0\left({}^{z_0}/_{z}\right)\varepsilon_{EPU} \quad (2)$$

Thus, the initial dielectric constant, when $z = z_0$, is:

$$\varepsilon_{r,i} = (1 - \varphi_0)\varepsilon_{air} + \varphi_0\varepsilon_{EPU} \quad (3)$$

The sensitivity is then shown to be:

$$S = \frac{({}^{\Delta C}/_{C_0})}{\Delta P} = \frac{\left[\left({}^{\varepsilon_r z_0}/_{\varepsilon_{r,i} z}\right) - 1\right]}{\Delta P} \quad (4)$$

This simple model quantitatively describes our anticipation that as z decreases, the dielectric lattice will transition from a two-phase structure (low EPU-to-air ratio) with high sensitivity, to behaving more like a solid (high EPU-to-air ratio) with low sensitivity. The theoretical responses in **Figure 2C(i), 2E(i), and 2F(i)** were calculated using experimental displacement and force data input into the combination of Eq. (1) and Eq. (2). Pressure values up to 300 kPa were used, after which the calculated capacitance began to deviate significantly from the experimental results.

The discrepancy at high pressures can potentially be explained by representing the capacitance of the lattice-based sensor as an equivalent system. C_{eq}, seen in Eq. (5), is equivalent to the two EPU base layers in series with $C_{biphasic}$, which is modelled as the capacitance of the EPU lattice struts in parallel with the air voids[38].

Eq. (1) is only valid at low pressures, i.e., when $t_{biphasic} >> t_{base}$, and consequently, $C_{biphasic} << C_{base}$ based on Eqs. (6)-(9). In this case, the biphasic term $\frac{1}{C_{biphasic}}$ is large and dictates the outcome of Eq. (5). As the lattice dielectric is highly compressed under large normal pressure, the instantaneous $C_{biphasic}$ value increases due to $t_{lattice}$ becoming thinner. Consequently, $\frac{1}{C_{biphasic}}$ becomes more comparable to $\frac{1}{C_{base,1}}$ and $\frac{1}{C_{base,2}}$ and longer dominates the behavior of C_{eq}. Thus, at high pressures, the effect of the base layers begins to influence the model, so Eq. (2) must be modified to account for this effect.

$C_{eq} =$

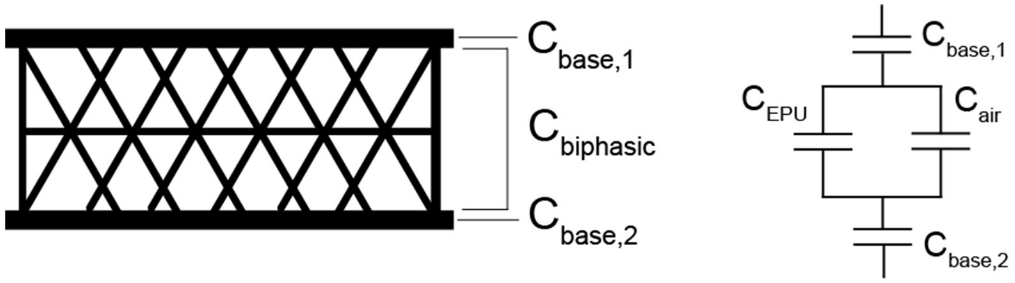

$$\frac{1}{\frac{1}{C_{base,1}}+\frac{1}{C_{biphasic}}+\frac{1}{C_{base,2}}} = \frac{1}{\frac{1}{C_{base,1}}+\frac{1}{C_{EPU}+C_{air}}+\frac{1}{C_{base,2}}} \quad (5)$$

The capacitances for each of the above terms are

$$C_{base,1} = \frac{\varepsilon_0 \varepsilon_{EPU} A}{t_{base}} \quad (6)$$

$$C_{EPU} = \frac{f_{EPU} \varepsilon_0 \varepsilon_{EPU} A}{t_{lattice}} \quad (7)$$

$$C_{air} = \frac{f_{air} \varepsilon_0 \varepsilon_{air} A}{t_{lattice}} \quad (8)$$

$$C_{base,2} = \frac{\varepsilon_0 \varepsilon_{EPU} A}{t_{base}} \quad (9)$$

Where f is the volume fraction of the component and t is the layer thickness ($t_{lattice,0} =$ 2.6 mm and $t_{base} = 0.5$ mm).

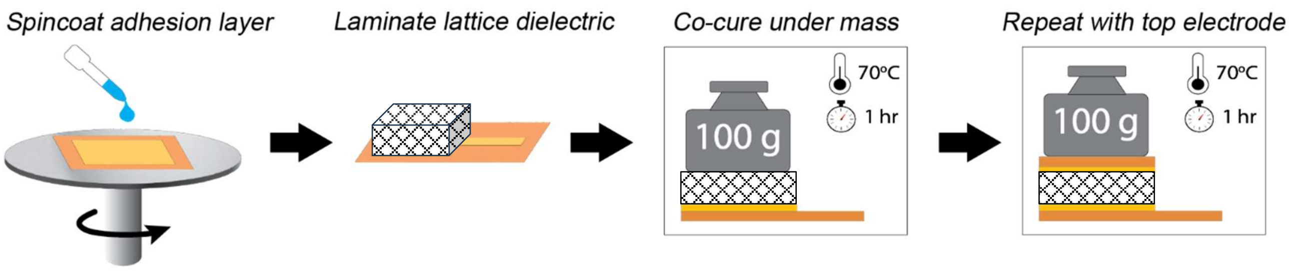

Fig. S1. Fabrication of single-pixel capacitive sensor. Schematic of the fabrication process for a single-pixel parallel-plate sensor with an EPU lattice dielectric layer and copper electrodes on flexible polyimide.

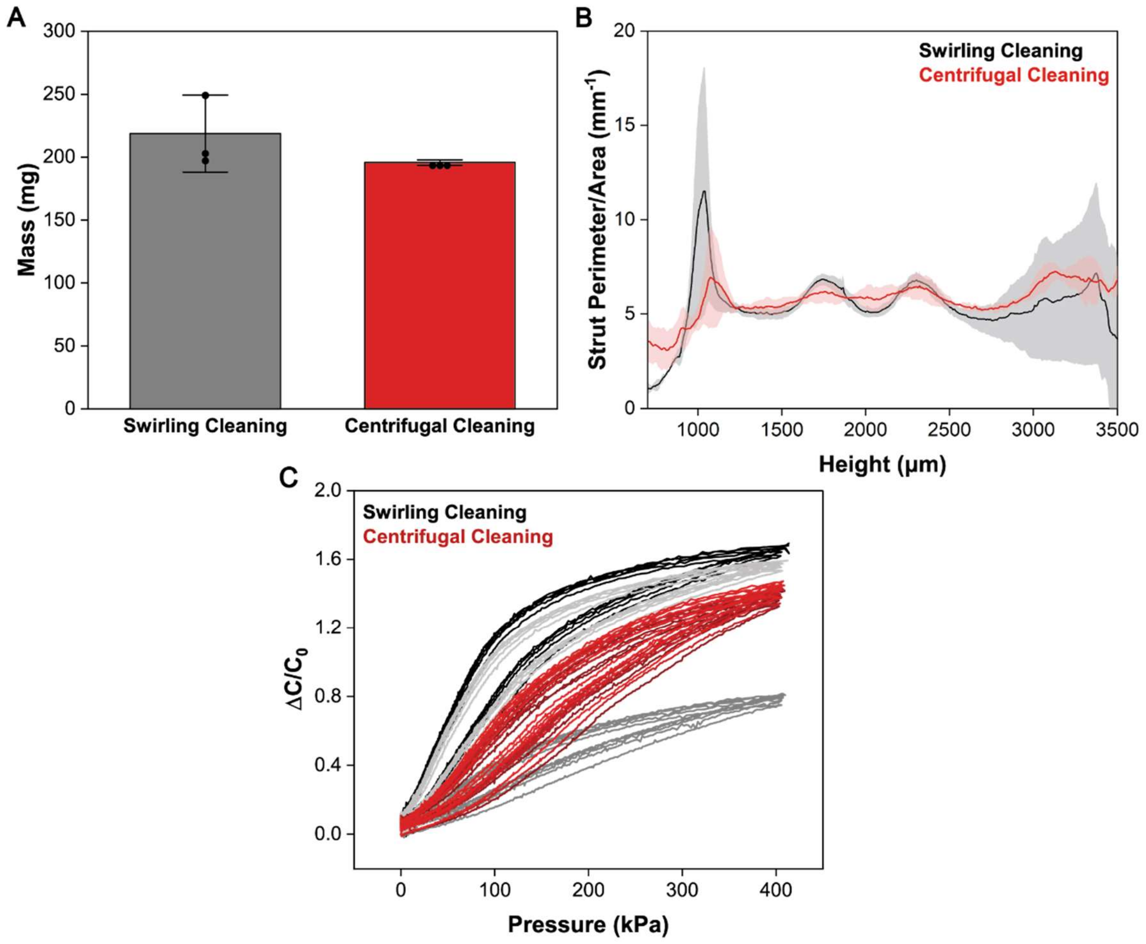

Fig. S2. Effect of cleaning of printed part on capacitive sensing reproducibility. (A) The mass of the lattices was chosen as a quality control parameter, as it represents the quantity of resin within each individual print. When compared to the typical cleaning technique by swirling in isopropyl alcohol (IPA), the novel centrifugal-based cleaning technique produced lattices of highly repeatable masses. Error bars are +/-s.d. for n = 3. (B) MicroCT analysis showed less variation in the cured polymeric structure through the height of the lattice. (C) A plot of the normalized change in capacitance proved the superiority of the centrifugal cleaning method in having three capacitive sensors with overlapping response, compared to the differences between devices made following the IPA swirling method. Each device was cycled three times.

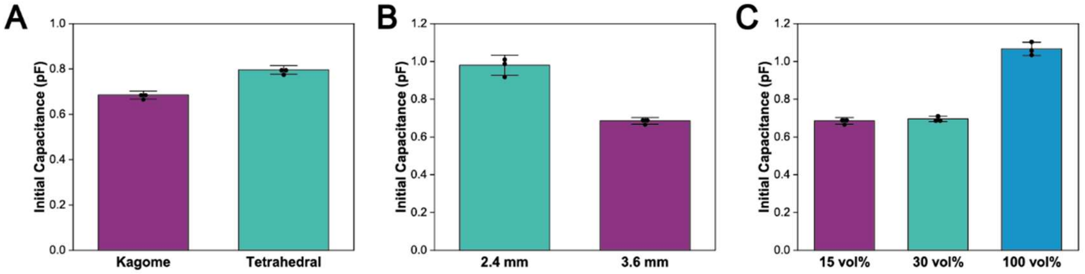

Fig. S3. **Initial capacitances of single-pixel sensors.** Initial capacitances of single-pixel sensors comparing different (A) lattice types (3.6 mm thickness, 15 vol%) (B) lattice thicknesses (kagome design, 15 vol%) and (C) lattice volume percentages (kagome design, 3.6 mm thickness). Error bars are +/-s.d. for n = 3.

The tetrahedral metastructure had a higher initial capacitance than the kagome (**Fig. S4A)**. This finding was unexpected, as the microstructural design was not considered in the capacitive model, Eq. (1). However, the lower initial capacitance could be an artifact of creep within the softer tetrahedral lattice under sustained force during co-curing in the oven for device fabrication, which may result in a slightly thinner dielectric layer.

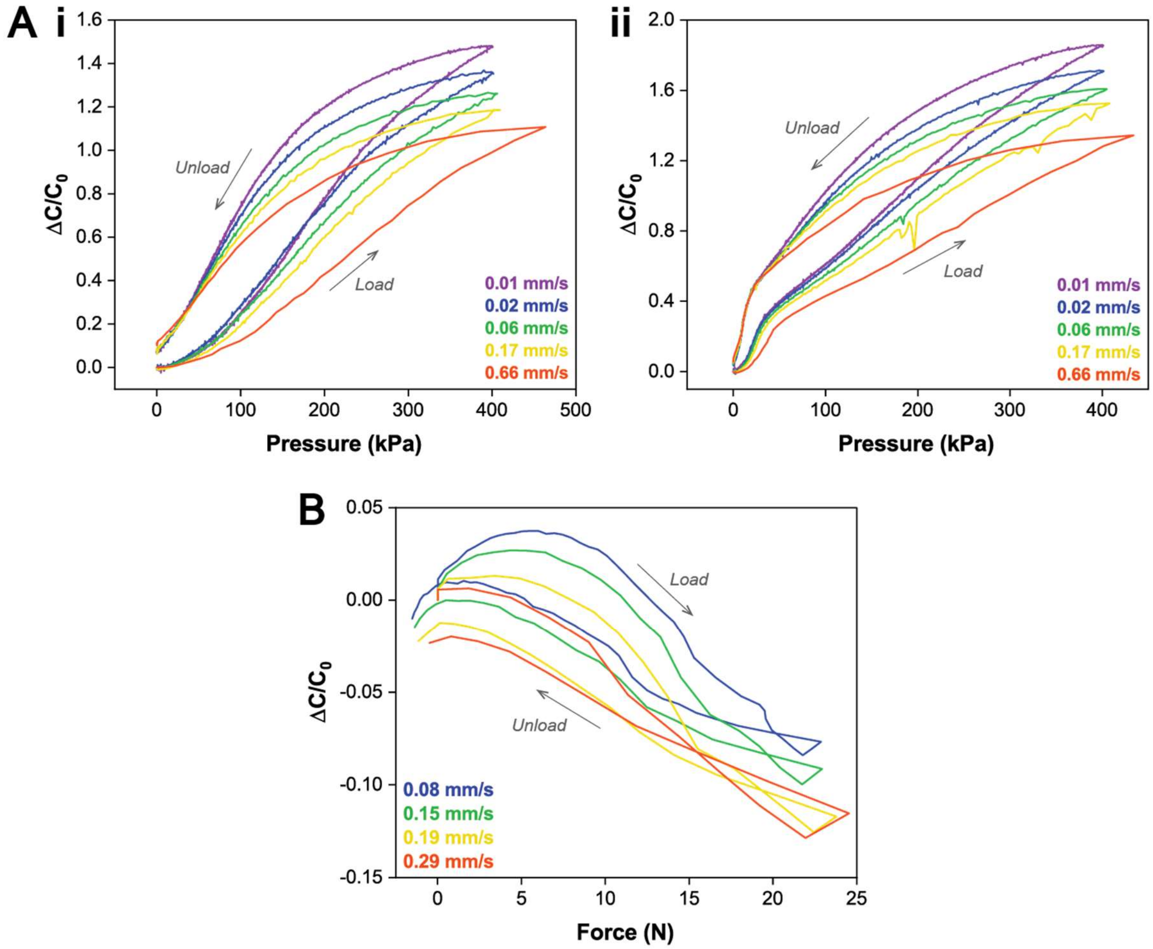

Fig. S4. Rate dependence of single pixel lattice sensors. (A) Representative plots showing the effect of normal pressure strain rate (mm/s) on $\Delta C/C_0$ vs. Pressure (kPa) of (i) kagome, 3.6 mm, 15 vol% and (ii) tetrahedral, 3.6 mm, 15 vol%. (B) Representative data of different shear rates (mm/s) on capacitive sensing of a tetrahedral, 3.6 mm, 15 vol% lattice-based sensor.

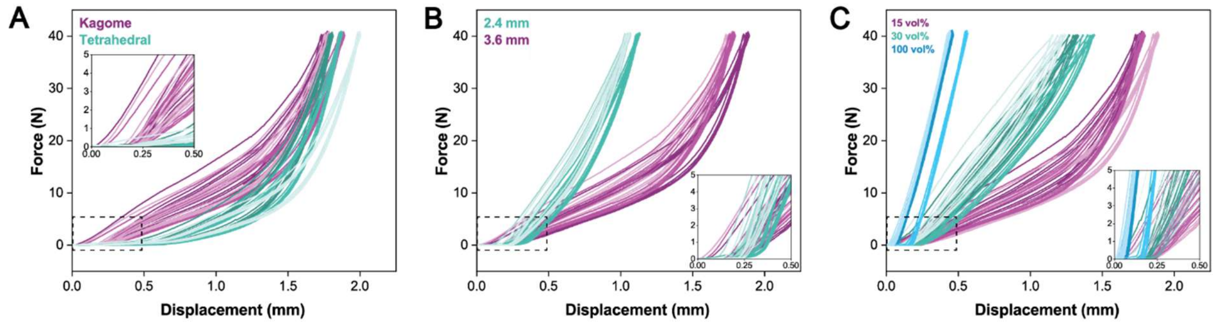

Fig. S5. Mechanical response of single-pixel lattice sensors. Force (N) versus displacement (mm) of lattices up to 400 kPa of normal pressure comparing different (A) lattice types (3.6 mm thickness, 15 vol%) (B) lattice thicknesses (kagome design, 15 vol%) and (C) lattice volume percentages (kagome design, 3.6 mm thickness). Each set has three samples that are cyclically loaded three times. The insets show zoomed-in data from 0-0.5 mm displacement and 0-5 N force.

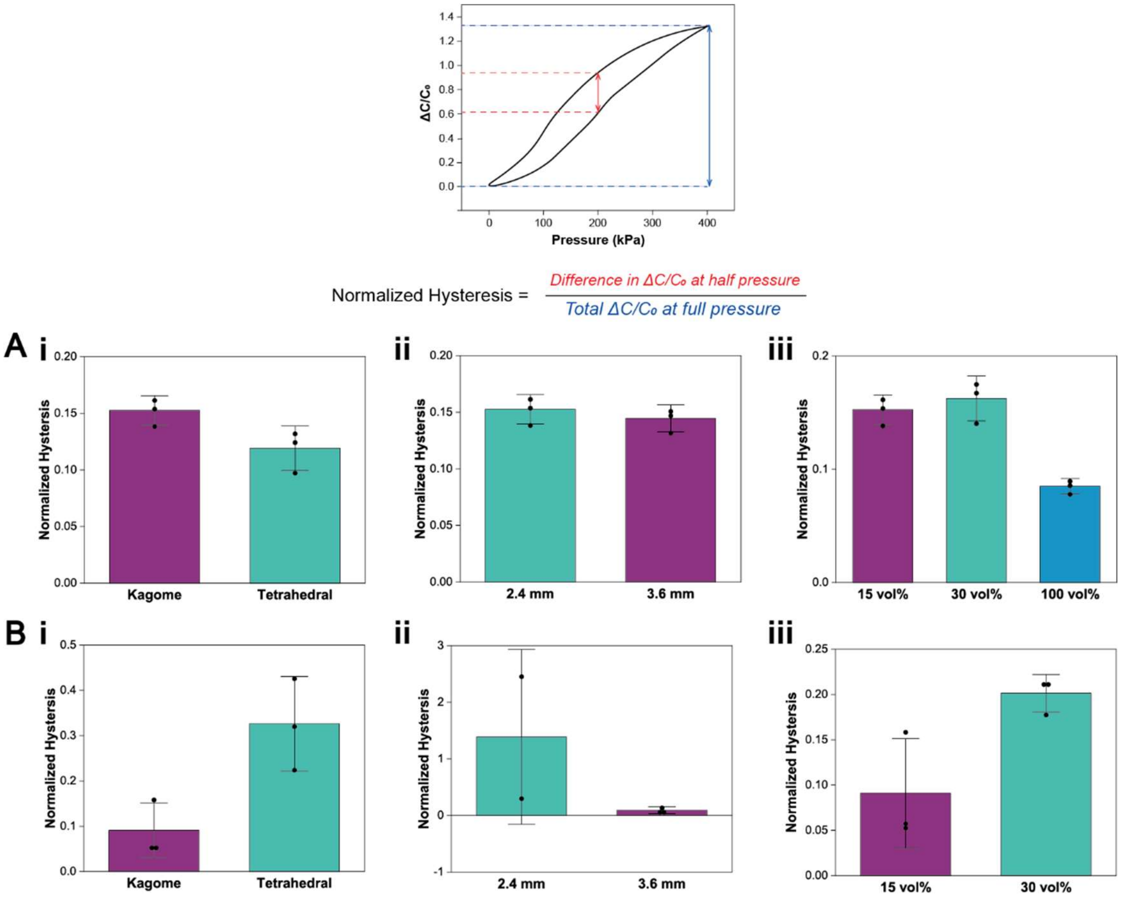

Fig. S6. Normalized hysteresis of single-pixel sensors. Normalized hysteresis equations and bar graphs showing the results for (A) normal pressure (B) shear force across different (i) lattice types (3.6 mm thickness, 15 vol%), (ii) thicknesses (kagome design, 15 vol%), and (iii) volume percentages (kagome design, 3.6 mm thickness). Error bars are +/- s.d. for n = 3.

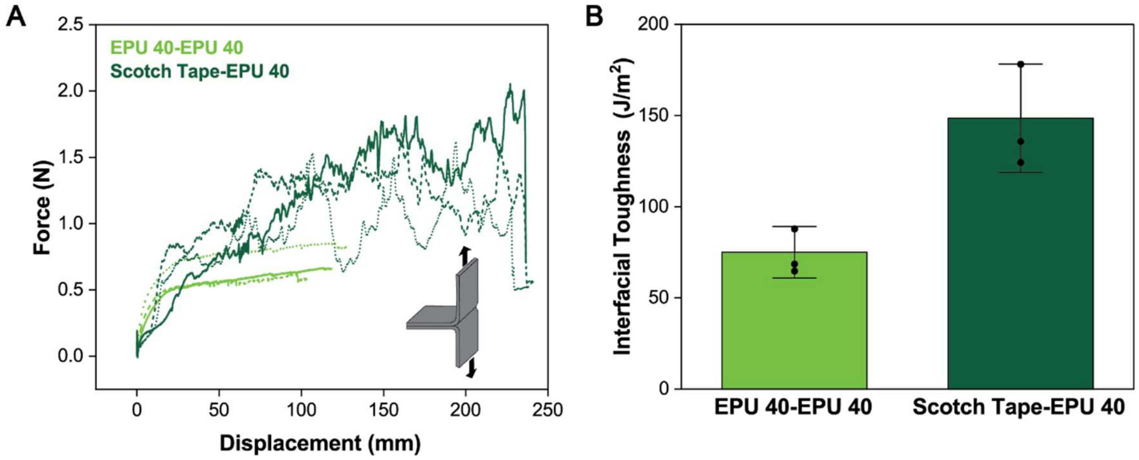

Fig. S7. 180º peel test showing differences in adhesion. (A) EPU 40-EPU 40 and scotch tape-EPU 40 and (B) the calculated interfacial toughness with error bars of +/- s.d. for n = 3. The interfacial toughness was calculated by doubling the average force plateau and dividing by the sample width.

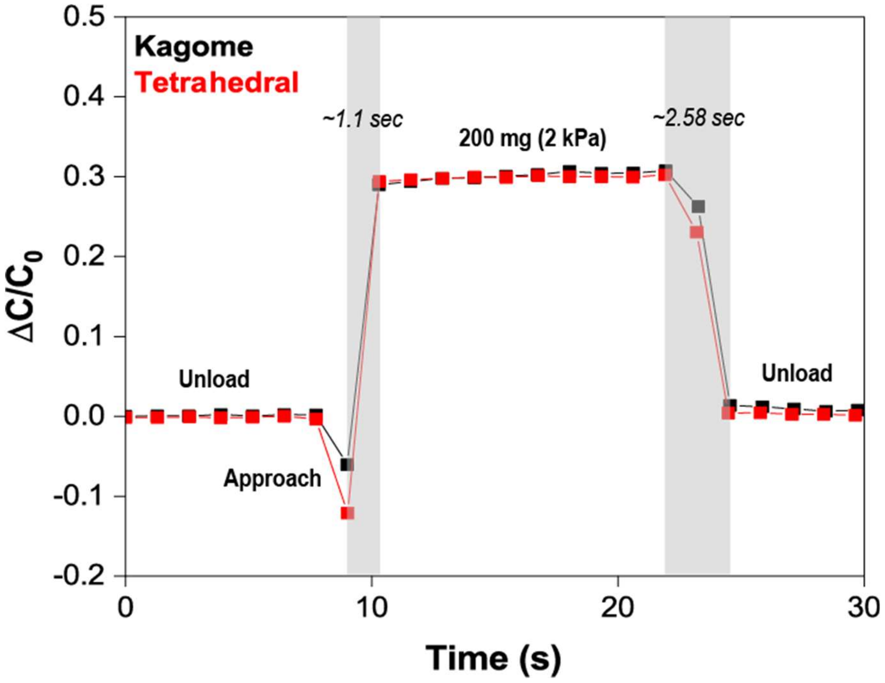

Fig. S8. Sensor response time. $\Delta C/C_0$ versus time (s) for kagome and tetrahedral 3.6 mm, 15 vol% lattices under 200 mg (2 kPa).

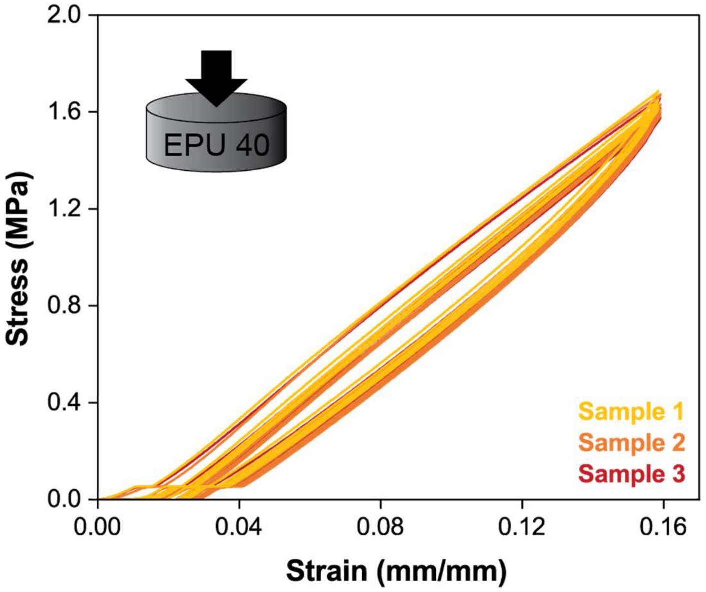

Fig. S9. Experimental bulk compression testing of EPU 40. Stress (kPa) versus strain (mm/mm) of three printed pucks of EPU 40 to understand the inherent hysteresis of the material.

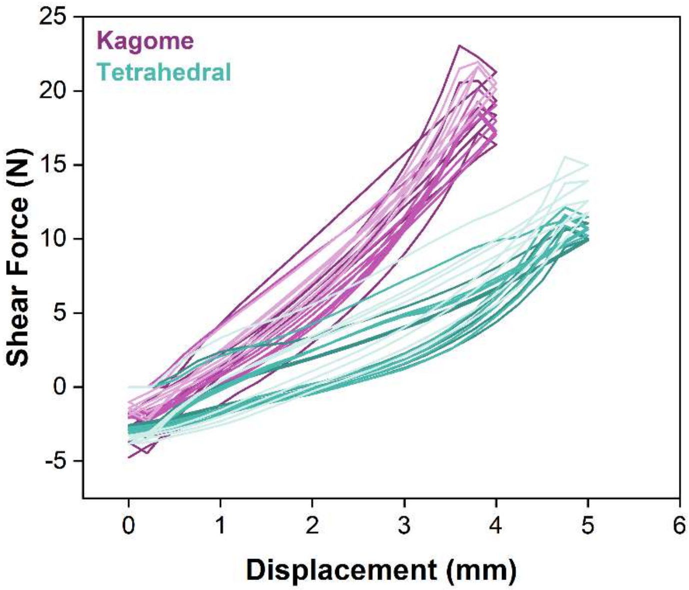

Fig. S10. Mechanical response of single-pixel sensors under shear force. Shear force (N) vs. displacement (mm) of kagome and tetrahedral lattice types with constant 3.6 mm thickness and 15 vol%. Each set had three samples that were cyclically loaded three times.

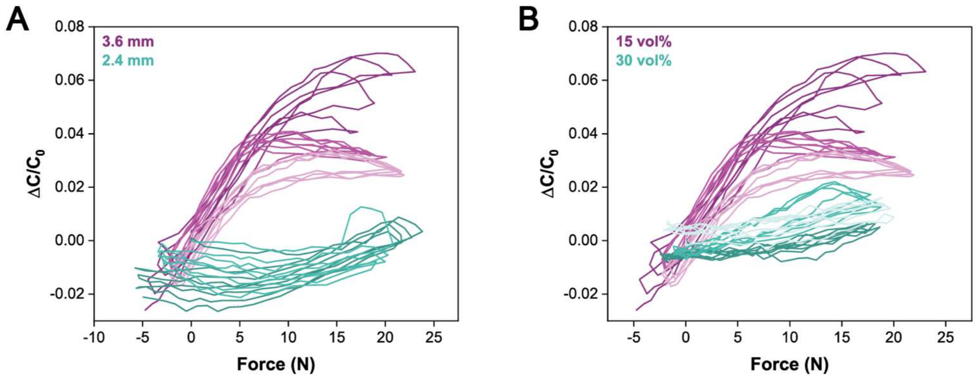

Fig. S11. Capacitive response under shear force of single-pixel sensors. Graphs showing $\Delta C/C_0$ versus shear force for (A) lattice thickness (kagome design, 15 vol%) and (B) lattice volume percentages (kagome design, 3.6 mm thickness). Neither 2.4 mm lattices nor 30 vol% lattices have shear sensing capability. Each set had three samples that were cyclically loaded three times. The 2.4 mm thickness and 30 vol% samples were tested at shear rates of 0.111 mm/s.

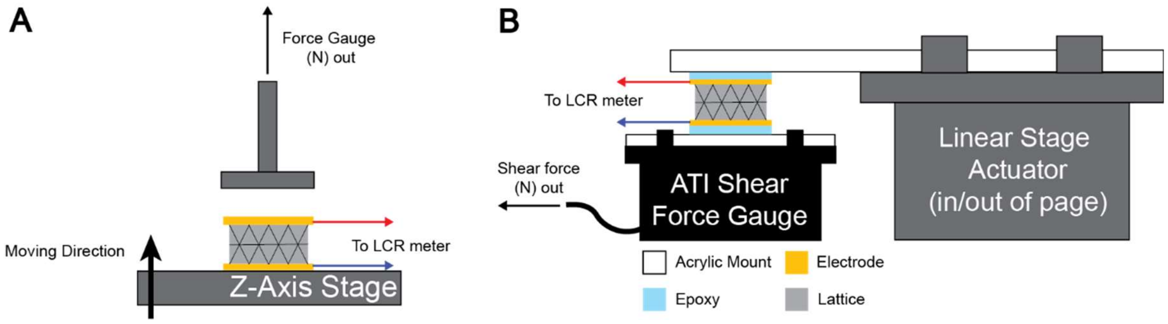

Fig. S12. Schematics of testing apparatuses and LCR meter readout. (A) Normal pressure and (B) Shear force.

Table S1. Comparison of lattice design parameters and resulting printed part. Lattice material-void volume percentages and strut widths are assessed.

Lattice Type	Lattice Thickness	Design Volume Percent	Printed Volume Percent	Design Strut Width	Printed Strut Width	Maximum Cell Size
	mm	*vol%*	*vol%*	*µm*	*µm*	*mm*
Kagome	2.4	15	20.3 ± 0.01	280	294	1.29
Kagome	3.6	15	19.5 ± 0.03	211	230	1.65
Kagome	3.6	30	27.4 ± 0.04	300	320	1.39
Tetrahedral	3.6	15	15.4 ± 0.05	207	210	1.32

Table S2. Comparison of multiaxial and lattice-based capacitive devices in literature that can sense both normal and shear forces. The metrics include dielectric microstructure, fabrication method and complexity, and sensitivities.

Ref.	Dielectric Microstructure	Normal Sensing?	Shear Sensing?	3D printing?	Fabrication Complexity	*Normal Pressure*		*Shear Force*	
						**Low-Pressure Sensitivity**	**High-Pressure Sensitivity**	**Low-Pressure Sensitivity**	**High-Pressure Sensitivity**
This work	Lattice	Yes	Yes	Yes	Low	0.0063 kPa^{-1}	0.0016 kPa^{-1}	-0.008 N^{-1}	-0.022 N^{-1}
34	Cellular	Yes	No	Yes	Low	1.23 kPa^{-1}	0.05 kPa^{-1}	-	-
35	Lattice	Yes	No	Yes	Low	-	-	-	-
2	Foam	Yes	Yes	No	Low	380 counts N^{-1}	-	x: 682, y: 1409 counts N^{-1}	-
29	Foam	Yes	Yes	No	Medium	0.378 kPa^{-1}	0.218 kPa^{-1}	-	-
31	Foam	Yes	Yes	No	High	0.04 kPa^{-1}	0.006 kPa^{-1}	0.1 kPa^{-1}	0.018 kPa^{-1}
30	Foam, Microfluidic Channel	Yes	Yes	No	High	2.77 $mNfF^{-1}$	-	0.23 $mNfF^{-1}$	-
1	Dome Microstructure	Yes	Yes	No	Medium	0.19 kPa^{-1}	0.001 kPa^{-1}	3.0 kPa^{-1}	-
33	Air gap	Yes	Yes	Yes	Low	2.3 pF/MPa	-	-	-
22	Air gap	Yes	Yes	No	Low	0.91 kPa^{-1}	0.02 kPa^{-1}	-	0.3 N^{-1}
23	Air gap	Yes	Yes	No	High	1.2 %/mN	-	x: 1.3, y: 1.2 %/mN	-
4	-	Yes	Yes	No	Medium	4 fF N^{-1}	-	3.3 fF N^{-1}	-
14	-	Yes	Yes	No	Low	0.70 kPa^{-1}	0.10 kPa^{-1}	-	-
